# National hepatitis B and C estimates for 2021: Measuring Canada’s progress towards eliminating viral hepatitis as a public health concern

**DOI:** 10.14745/ccdr.v51i67a02

**Published:** 2025-07-01

**Authors:** Simone Périnet, Anson Williams, Laurence Campeau, Janelle Elliott, Fan Zhang, Qiuying Yang, Joseph Cox, Karelyn Davis, Jordan J Feld, Marina B Klein, Nadine Kronfli, Mia J Biondi, Peter K Daley, Nashira Popovic

**Affiliations:** 1 Centre for Communicable Diseases and Infection Control, Public Health Agency of Canada, Ottawa, ON; 2 Department of Epidemiology, Biostatistics and Occupational Health, McGill University, Montréal, QC; 3 Toronto Centre for Liver Disease, University Health Network, University of Toronto, Toronto, ON; 4 Research Institute of the McGill University Health Centre, Montréal, QC; 5 CIHR Pan-Canadian Network for HIV and STBBI Clinical Trials Research (CTN+), Montréal, QC; 6 Department of Medicine, Division of Infectious Diseases and Chronic Viral Illness Service, McGill University, Montréal, QC; 7 Centre for Outcomes Research and Evaluation, Research Institute of the McGill University Health Centre, Montréal, QC; 8 School of Nursing, York University, Toronto, ON; 9 Memorial University of Newfoundland, St. John’s, NL

**Keywords:** viral hepatitis, hepatitis B, hepatitis C, prevalence, incidence, key populations, estimations, Canada

## Abstract

**Background:**

Hepatitis B virus (HBV) and hepatitis C virus (HCV) infections are major causes of morbidity and mortality worldwide. Measuring the epidemiological burden of HCV and HBV in Canada is essential to measure progress towards global elimination targets and to ultimately eliminate viral hepatitis as a public health concern.

**Objective:**

This study aimed to provide the first national estimates of HBV prevalence and unawareness, and to update estimates of HCV incidence, prevalence, and unawareness in the general population and key populations in Canada for 2021. Progress towards elimination targets for 2025, namely incidence, awareness, mortality, and HBV vaccination, was also assessed.

**Methods:**

A combination workbook method and mathematical modelling was used to estimate the prevalence and unawareness of chronic hepatitis B (CHB), prevalence and incidence of anti-HCV antibodies, and the prevalence and unawareness of chronic hepatitis C (CHC).

**Results:**

The estimated prevalence of CHB was 0.68% (plausible range: 0.40%–0.97%) or 262,000 (152,000–371,000) people in the general population, of whom 42.5% (33.9%–51.0%) were unaware of their infection. Immigrants from countries where HBV is common had the highest prevalence at 4.2% (1.9%–5.6%). An estimated 8,212 new HCV infections occurred in 2021, and the estimated prevalence of CHC was 0.56% (0.15%–0.97%) or 214,000 (58,500–369,000) people, of whom 41.5% (34.3%–48.8%) were unaware of their infection. People who inject drugs had the highest prevalence and largest proportion who were unaware at 36.9% (12.6%–55.1%) and 49.9% (29.0%–70.2%), respectively.

**Conclusion:**

While the overall viral hepatitis burden is low in the general Canadian population, these estimates indicate that certain populations and communities remain disproportionately affected. Although Canada has met some of the 2025 targets, more work is needed. To this end, efforts to obtain and standardize provincial and national data will be required to measure progress towards all elimination targets.

## Introduction

Hepatitis B virus (HBV) and hepatitis C virus (HCV) infections are sexually transmitted and blood-borne infections (STBBI) leading to chronic liver disease with a risk of progression to cirrhosis, liver failure and liver cancer (1), despite the availability of effective treatment and HBV vaccines. Following HBV infection, approximately 90% of newborns, 20%–30% of children younger than five years, and 5%–10% of adults younger than 50 years develop chronic hepatitis B (CHB) ((2,3)) which may cause progressive liver injury. Suppressive treatment is available ((3)) and reduces the risk of liver-related outcomes but must be taken long-term and rarely leads to clearance of the infection. People exposed to and infected with HCV develop antibodies that remain detectable regardless of spontaneous clearance or successful treatment. Detection of viral RNA is necessary to diagnose chronic hepatitis C (CHC) infection, defined as persistence of viremia beyond six months ((4)). More than 95% of people who take direct-acting antivirals (DAAs) treatment achieve a sustained virological response 12 weeks after the end of treatment and are therefore considered cured ((5)). Treatment is well tolerated and recommended for all people living with CHC ((6)).

Key guiding documents identify certain populations and communities that are disproportionately impacted by HBV and HCV (7–10), including immigrants from countries where HBV or HCV is common, gay, bisexual and other men who have sex with men (GBMSM), incarcerated people, people who inject drugs (PWID), First Nations, Inuit and Métis, and adults in the 1945–1975 birth cohort. Canada is committed to achieving the global targets in support of eliminating viral hepatitis as a public health concern ((8,11)). Global targets include a reduction of incidence and mortality, an increase in the proportion of people living with CHC or CHB who are diagnosed and treated, and an increase of HBV vaccine coverage.

This paper provides updated national estimates of HCV incidence, prevalence, awareness and treatment, as well as the first national estimates of HBV prevalence and awareness, for 2021 ((9)). These estimates are reported for the general population, as well as for key populations. Finally, Canada’s progress towards some of the 2025 global viral hepatitis elimination targets are discussed.

## Methods

A combination of a modified workbook method and mathematical modelling was used to estimate the prevalence of CHB and the proportion of people living with CHB who were unaware of their infection, and to estimate the prevalence of anti-HCV antibodies and CHC and the proportion of people living with CHC who were unaware of their infection. Incidence for HCV was estimated using mathematical modelling. Mortality, HBV vaccination and HCV treatment were estimated using administrative data. Rates were calculated using national population estimates ((12)).

### Hepatitis B

#### Systematic review

A systematic search for literature published between January 1, 2016 and March 31, 2023, on prevalence and unawareness of HBV infection in Canada was conducted in the MEDLINE, Embase and Scopus databases and yielded an initial 355 records, with an additional 40 records identified through other sources. Using a previously described method ((13)), 14 records were selected to consider in the workbook method, in addition to unpublished data from organizations and researchers.

#### Mathematical modelling

National CHB modelling was described elsewhere ((14)) and used data from the Canadian Notifiable Disease Surveillance System (CNDSS), extracted in July 2023 for all provinces and territories, as input into the model.

#### Modified workbook

A modified workbook method ((13)) was used to estimate the prevalence of CHB infection (using the hepatitis B surface antigen (HBsAg) as a proxy for CHB, where applicable), and its proportion unaware, for the general population and for key populations informed by an environmental scan. Each measure extracted from the systematic search records were classified as “underestimate,” “appropriate estimate” or “overestimate” based on a review of the methodology of each study. The underestimates and overestimates were used as plausible ranges. When measures from multiple records were available, evidence was ranked to prioritize representative data (e.g., national surveys) over data of lower representativity (e.g., local studies) to determine each plausible range. Where possible, results from comparable studies were averaged. Point estimates were calculated as the midpoint between bounds, except when representative data (“appropriate estimates”) were used as the point estimate. In instances where only one representative estimate was available, the plausible range is the 95% confidence interval, computed as needed using the Wilson method ((15)). In instances where there was one data source of low representativity or no data, “insufficient data” was indicated. Proportions ≤1 were rounded to two decimal places, larger proportions were rounded to one decimal place, and absolute numbers were rounded to three significant digits.

#### Administrative data: Vaccination and mortality

Vital statistics data ((16)) were used to estimate the proportion of all live births in Canada that occurred in provinces and territories offering universal birth dose vaccination. Vaccination coverage was taken from the *Childhood National Immunization Coverage Survey* report ((17)).

Vital statistics data were used to directly estimate the number of deaths in 2021 for which HBV (ICD-10 codes B16.0, B16.1, B16.2, B16.9, B17.0, B18.0, and B18.1) was identified as the underlying cause or one of the other 19 contributing causes of death ((18)).

### Hepatitis C

#### Systematic review

A search on prevalence, incidence and unawareness of infection in Canada was updated with records published between July 1, 2021 and March 31, 2023, yielding 187 records, with an additional 28 records found outside of the search. A total of 31 records were selected for consideration using the workbook method, in addition to the 22 records identified and used through the 2019 estimates process ((13)) and unpublished data from organizations and researchers.

#### Mathematical modelling

Back-calculation mathematical modelling ((7,19)) with least square method ((20)) for 2020–2021 pandemic adjustment was used to estimate HCV incidence and as input for the anti-HCV antibodies prevalence estimates. Data from a July 2023 CNDSS extraction for British Columbia, Alberta, Saskatchewan, Ontario, Québec and the Yukon were used as input and model outputs were projected to Canadian population.

#### Modified workbook

Using methods described in the HBV section, this study estimated the prevalence of anti-HCV antibodies (as a marker of current or past infection) as well as the prevalence of CHC infection (using detection of HCV RNA as a proxy where applicable) and its proportion unaware, for the general population and for key populations, based on priority populations identified in the blueprint to inform hepatitis C elimination efforts in Canada ((7)).

#### Administrative data: Treatment and mortality

Licensed data were obtained from IQVIA Solutions Canada (IQVIA) to estimate the number of people treated for HCV in Canada between 2012 and 2021. The 2012–2016 estimates were produced by the British Columbia Centre for Disease Control using Compuscript data from IQVIA, and the 2017–2021 estimates were projected patient counts computed by IQVIA using the GPM Custom Solutions information service.

Vital statistics data were used to directly estimate the number of deaths in 2021 for which HCV (ICD-10 codes B17.1 and B18.2) was identified as an underlying cause or one of the other 19 contributing causes of death ((18)).

## Results

### Chronic hepatitis B prevalence

The prevalence of CHB in the general population was estimated at 0.68% (plausible range: 0.40%–0.97%) or 262,000 (152,000–371,000) people at the end of 2021. Among key populations, the highest estimated prevalence was 4.2% (1.9%–5.6%) among immigrants from countries where HBV is common (≥2% HBsAg prevalence), followed by GBMSM at 1.4% (0.8%–2.1%) and people incarcerated in federal prisons (**Table 1**). Additional data are needed to estimate the prevalence in other key populations.

**Table 1:** Estimated chronic hepatitis B prevalence for the general population and key populations, Canada, 2021

Population	Population size estimate	Prevalence (%)			Estimated number of people living with CHB			Reference(s)
		Point estimate	Lower bound	Upper bound	Point estimate	Lower bound	Upper bound	
General population	38,239,864	0.68%	0.40%	0.97%	262,000	152,000	371,000	((12,14,21–24))
Immigrants from countries where HBV is common (HBsAg ≥2%)	5,599,485	4.2%	1.9%	5.6%	237,000	106,000	312,000	((21,25,26))
GBMSM	669,613	1.4%	0.8%	2.1%	9,310	5,360	14,100	((27,28)) Unpublished data from the Engage cohort study, 2017–2019
People incarcerated in federal prisons^a^	12,405	0.28%	0.19%	0.41%	35	24	51	Unpublished data from Correctional Service Canada, 2021 (*personal communication, 2024*)
People incarcerated in provincial prisons^b^	Insufficient information							
PWID	Insufficient information							
First Nations	Insufficient information							
Inuit	Insufficient information							
Métis	Insufficient information							

Abbreviations: CHB, chronic hepatitis B; HBsAg, hepatitis B surface antigen; HBV, hepatitis B virus; GBMSM, gay, bisexual and other men who have sex with men; PWID, people who inject drugs

^a^ Who were sentenced for two years or more

^b^ Who were sentenced for less than two years

### Unawareness of chronic hepatitis B infection

The proportion of people living with CHB who were unaware of their infection was estimated at 42.5% (33.9%–51.0%), or 111,000 (88,700–134,000) people (**Table 2**). Additional data are needed to estimate unawareness in key populations.

**Table 2:** Estimated number and proportion of people living with chronic hepatitis B who were unaware of their infection for the general population and key populations, Canada, 2021

Population	Estimated number of people living with CHB	Proportion who were unaware (%)			Estimated number of people who were unaware			Reference(s)
		Point estimate	Lower bound	Upper bound	Point estimate	Lower bound	Upper bound	
General population	262,000	42.5%	33.9%	51.0%	111,000	88,700	134,000	((14,21))
Immigrants from countries where HBV is common (HBsAg ≥2%)	Insufficient information							
GBMSM	Insufficient information							
People incarcerated in federal prisons^a^	Insufficient information							
People incarcerated in provincial prisons^b^	Insufficient information							
PWID	Insufficient information							
First Nations	Insufficient information							
Inuit	Insufficient information							
Métis	Insufficient information							

Abbreviations: CHB, chronic hepatitis B; HBsAg, hepatitis B surface antigen; HBV, hepatitis B virus; GBMSM, gay, bisexual and other men who have sex with men; PWID, people who inject drugs

^a^ Who were sentenced for two years or more

^b^ Who were sentenced for less than two years

### Hepatitis B vaccination

In the seven jurisdictions with a three-dose program for infants, the HBV vaccine coverage among two-year-old children was 82.6% (79.7%–85.1%) in 2021. The national HBV vaccine coverage among 14-year-old adolescents was 89.0% (86.3%–91.2%) in 2021 ((17)).

Three (New Brunswick, Northwest Territories and Nunavut) out of 13 provinces and territories have universal HBV vaccination programs initiated at birth, and 7,690 live births occurred in these jurisdictions between July 1, 2021 and June 30, 2022, representing 2% of live births in Canada during that period ((16)).

### Hepatitis B-related mortality

**Figure 1:**
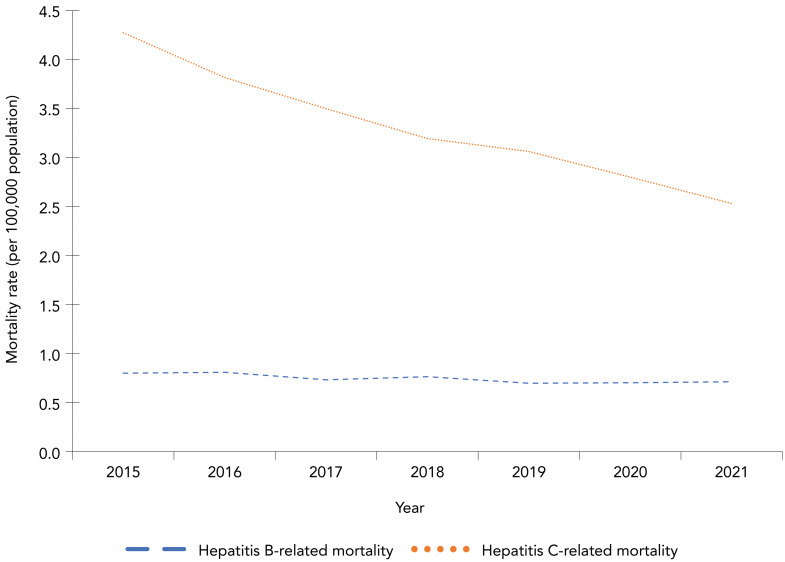
Crude annual hepatitis B-related and hepatitis C-related mortality rates (per 100,000 population), Canada, 2015–2021

In 2021, HBV was identified as a contributing cause for 274 deaths for a crude mortality rate of 0.72 per 100,000 population. Between 2015 and 2021, the annual mortality rate for HBV-related deaths was relatively stable (**Figure 1**).

### Hepatitis C incidence

**Figure 2:**
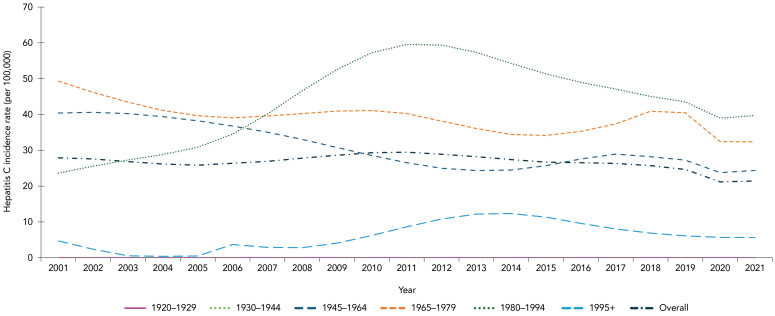
Estimated annual hepatitis C incidence rates (per 100,000 population), by birth cohort and overall, Canada, 2001–2021

An estimated 8,212 seroconversions (representing new HCV infections) occurred in 2021 in Canada, corresponding to an annual incidence rate of 21.47 per 100,000 population. The overall incidence rate has been slowly declining since 2013 (**Figure 2**). When grouped by birth cohort, as a proxy for established generations, those born between 1980 and 1994 had the highest incidence in 2021 (Figure 2) with 3,179 estimated seroconversions, for a rate of 39.76 per 100,000 population (data not shown).

### Hepatitis C prevalence

The estimated prevalence of anti-HCV antibodies in Canada was 0.99% (0.68%–1.30%) or 378,000 (258,000–497,000) people who were ever infected with HCV at the end of 2021. Among key populations, the highest estimated prevalence was among PWID (within 12 months) at 64.2% (36.7%–91.8%), followed by those who have ever injected drugs (35.4%; 9.3%–61.5%). In absolute numbers, adults in the 1945–1975 birth cohort represented the population with the largest number of people ever infected at 270,000 (157,000–383,000) (**Table 3**).

**Table 3:** Estimated anti-hepatitis C virus antibodies prevalence for the general population and key populations, Canada, 2021

Population	Population size estimate	Prevalence (%)			Estimated number of people with current or past HCV infection			Reference(s)
		Point estimate	Lower bound	Upper bound	Point estimate	Lower bound	Upper bound	
General population	38,239,864	0.99%	0.68%	1.3%	378,000	258,000	497,000	((12,21,29–31)) Unpublished data from PHAC, 2021 (this study)
PWID, within 12 months	100,300	64.2%	36.7%	91.8%	64,400	36,800	92,100	((32–35)) Unpublished data from the Virtual Cascade of Care Cohort study, 2018–2019 (*personal communication, Stine Høj, 2023*) Unpublished data from the HEPCO study, 2022–2023 (*personal communication, Sarah Larney, 2024*)
People who have ever injected drugs	388,400	35.4%	9.3%	61.5%	137,000	36,100	239,000	((21,33,35–37)) Unpublished data from the Virtual Cascade of Care Cohort study, 2018–2019 (*personal communication, Stine Høj, 2023*)
People incarcerated in provincial prisons^a^	18,950	14.2%	9.5%	19.0%	2,700	1,790	3,600	((38–41)) Unpublished data from Alberta Health Services, 2023 (*personal communication, Kaylee Goralcyk, 2024*)
People incarcerated in federal prisons^b^	12,405	11.1%	10.2%	12.0%	1,370	1,260	1,490	Unpublished data from Correctional Service Canada, 2021 (*personal communication, 2024*)
First Nations	1,048,400	8.0%	3.5%	12.5%	84,000	36,600	131,000	((42–44)) Unpublished data from the CHMS, Statistics Canada, 2014–2015
Immigrants from countries where hepatitis C is common (≥2% seroprevalence)	1,276,405	4.0%	2.9%	6.7%	51,500	36,700	85,300	((25))
GBMSM	669,613	3.0%	0.82%	5.2%	20,100	5,490	34,800	((27,45,46)) Unpublished data from the Engage cohort study, 2017–2019
Adults in the 1945–1975 birth cohort	14,267,340	1.9%	1.1%	2.7%	270,000	157,000	383,000	((12,21,29–31,36,47)) Unpublished data from PHAC, 2021 (this study)
Inuit	Insufficient information							
Métis	Insufficient information							

Abbreviations: CHMS, Canadian Health Measures Survey; HCV, hepatitis C virus; HEPCO, Hepatitis Cohort; GBMSM, gay, bisexual and other men who have sex with men; PHAC, Public Health Agency of Canada; PWID, people who inject drugs

^a^ Who were sentenced for two years or more

^b^ Who were sentenced for less than two years

The prevalence of CHC in Canada was estimated at 0.56% (0.15%–0.97%) or 214,000 (58,500–369,000) people living with CHC at the end of 2021. Among key populations, PWID (within 12 months) and those who have ever injected drugs had the highest estimated prevalence of CHC at 36.9% (12.6%–55.1%) and 18.1% (5.7%–30.5%), respectively. In absolute numbers, adults in the 1945–1975 birth cohort had the largest number of people living with CHC at 157,000 (30,000–284,000) (**Table 4**).

**Table 4:** Estimated chronic hepatitis C prevalence for the general population and key populations, Canada, 2021

Population	Population size estimate	Prevalence (%)			Estimated number of people living with CHC			Reference(s)
		Point estimate	Lower bound	Upper bound	Point estimate	Lower bound	Upper bound	
General population	38,239,864	0.56%	0.15%	0.97%	214,000	58,500	369,000	((12,21,30,31,48–52))
PWID, within 12 months	100,300	36.9%	12.6%	55.1%	37,000	12,600	55,300	((32–35,53,54)) Unpublished data from the HEPCO study, 2022–2023 (*personal communication, Sarah Larney, 2024*) Unpublished data from the Virtual Cascade of Care Cohort study, 2018–2019 (*personal communication, Stine Høj, 2023*)
People who have ever injected drugs	388,400	18.1%	5.7%	30.5%	70,100	21,900	118,000	((21,35)) Unpublished data from the Virtual Cascade of Care Cohort study, 2018–2019 (*personal communication, Stine Høj, 2023*)
People incarcerated in provincial prisons^a^	18,950	5.1%	4.4%	5.7%	957	834	1,080	((38,40,41)) Unpublished data from Alberta Health Services, 2023 (*personal communication, Kaylee Goralcyk, 2024*)
First Nations	1,048,400	3.3%	1.5%	5.0%	34,300	15,800	52,800	((42–44,55,56))
People incarcerated in federal prisons^b^	12,405	3.2%	2.7%	3.7%	396	335	464	Unpublished data from Correctional Service Canada, 2021 (*personal communication, 2024*)
Immigrants from countries where hepatitis C is common (≥2% seroprevalence)	1,276,405	2.2%	1.6%	3.5%	28,100	19,800	44,600	((25))
Adults in the 1945–1975 birth cohort	14,267,340	1.1%	0.21%	2.0%	157,000	30,000	284,000	((12,21,48,49,57))
GBMSM	669,613	0.94%	0.18%	1.7%	6,290	1,210	11,400	((27,45,46)) Unpublished data from the Engage cohort study 2017–2019
Inuit	Insufficient information							
Métis	Insufficient information							

Abbreviations: CHC, chronic hepatitis C; HEPCO, Hepatitis Cohort; GBMSM, gay, bisexual and other men who have sex with men; PWID, people who inject drugs

^a^ Who were sentenced for two years or more

^b^ Who were sentenced for less than two years

### Unawareness of chronic hepatitis C infection

The proportion of people living with CHC who were unaware of their infection was estimated at 41.5% (34.3%–48.8%) or 104,000 people. Among key populations, the largest proportion was among PWID at 49.9% (29.0%–70.2%), followed by people incarcerated in federal prisons at 32.7% (28.2%–37.5%). In absolute numbers, adults in the 1945–1975 birth cohort had the largest number of people unaware of their infection at 45,200 (24,700–65,800) (**Table 5**). Additional data are needed to estimate unawareness in other key populations.

**Table 5:** Estimated number and proportion of people living with chronic hepatitis C who were unaware of their infection, for the general population and key populations, Canada, 2021

Population	Estimated number of people living with CHC	Proportion who were unaware (%)			Estimated number of people living with CHC who are unaware			Reference(s)
		Point estimate	Lower bound	Upper bound	Point estimate	Lower bound	Upper bound	
General population	214,000	41.5%	34.3%	48.8%	104,000	73,300	104,000	((21,48,49))
PWID, within 12 months	37,000	49.9%	29.0%	70.2%	18,500	10,700	26,000	((32))
People who have ever injected drugs	Insufficient information							
People incarcerated in provincial prisons^a^	Insufficient information							
First Nations	Insufficient information							
People incarcerated in federal prisons^b^	396	32.7%	28.2%	37.5%	129	112	148	Unpublished data from Correctional Services Canada, 2021 (*personal communication, 2024*)
Adults in the 1945–1975 birth cohort	157,000	28.9%	15.7%	41.9%	45,200	24,700	65,800	((21,48,49,58))
Immigrants from countries where hepatitis C is common (≥2% seroprevalence)	Insufficient information							
Inuit	Insufficient information							
Métis	Insufficient information							
GBMSM	Insufficient information							

Abbreviations; CHC, chronic hepatitis C; GBMSM, gay, bisexual and other men who have sex with men; PWID; people who inject drugs

^a^ Who were sentenced for two years or more

^b^ Who were sentenced for less than two years

### Hepatitis C treatment

**Figure 3:**
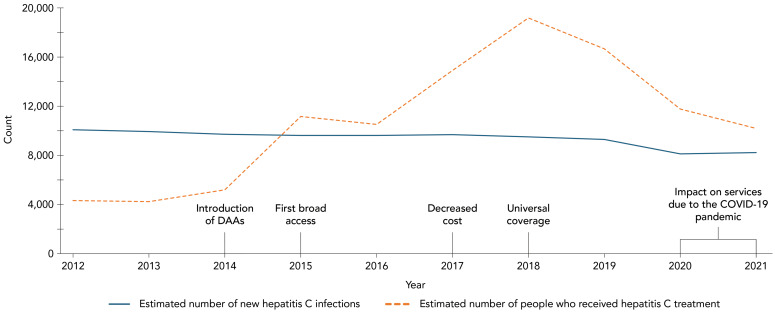
Estimated annual number of people living with chronic hepatitis C who were treated and estimated annual number of new hepatitis C infections, Canada, 2012–2021 Abbreviation: DAAs, direct-acting antivirals

It is estimated that, between 2012 and 2021, 108,000 people living with CHC received treatment. Since the introduction of highly effective DAAs in Canada in 2014, approximately 99,400 people were treated. Between 2015 and 2021, but drastically starting in 2017, the estimated number of people treated annually surpassed the estimated number of new infections annually (**Table 6**, **Figure 3**).

**Table 6:** Estimated number of new hepatitis C virus infections and estimated number of people who received hepatitis C virus treatment by year, Canada, 2012–2021

Year	Estimated number of new HCV infections	Estimated number of people who received HCV treatment
2012	10,075	4,370
2013	9,912	4,221
2014	9,690	5,147
2015	9,557	11,138
2016	9,580	10,496
2017	9,673	14,887
2018	9,506	19,155
2019	9,298	16,619
2020	8,086	11,774
2021	8,212	10,155

Abbreviation: HCV, hepatitis C virus

### Hepatitis C-related mortality

In 2021, HCV was identified as a contributing cause for 972 deaths for a crude mortality rate of 2.54 per 100,000 population. Between 2010 and 2021, the annual mortality rate decreased, starting in 2016 (Figure 1).

### Progress towards viral hepatitis elimination

**Table 7** summarises the intermediary global targets for 2025 in relation to the 2021 estimates. For HBV, these estimates suggest that, as of 2021, Canada was on track to meet or had met three of the seven targets for 2025. For HCV, these estimates suggest that, as of 2021, Canada was on track to meet or had met three of the eight targets for 2025.

**Table 7:** Progress towards viral hepatitis elimination targets for 2025 outlined in the Global Health Sector Strategy 2022–2030, Canada, 2021

Indicator	2025 target ((11))	2021 estimates	Reference(s)
**Hepatitis B**			
Hepatitis B surface antigen prevalence among children 0–4 years old	0.5%	Additional data needed	
Number of new hepatitis B infections per year	11/100,000 population	Additional data needed	
Number of people dying from hepatitis B per year	7/100,000 population	0.72/100,000 population	This study using Vital statistics data ((18))
Percentage of newborns who have benefitted from a timely birth dose of hepatitis vaccine and from other interventions to prevent the vertical (mother-to-child) transmission of hepatitis B virus	70%	Less than 2%	This study using Vital statistics data ((18))
Hepatitis B vaccine coverage among children (third dose)^a^	90%	89.0% among 14-year-olds, 1 or more dose	((17))
Percentage of people living with hepatitis B diagnosed	60%	57.5%	This study
Percentage of people living with hepatitis B diagnosed and treated	50%	Additional data needed	
**Hepatitis C**			
Number of new hepatitis C infections per year	13/100,000 population	21.47/100,000 population	This study
Number of new hepatitis C infections among PWID per year	3/100	Additional data needed	
Number of people dying from hepatitis C per year	3/100,000 population	2.54/100,000 population	This study using Vital statistics data ((18))
Number of needles and syringes distributed per PWID	200	Additional data needed	
Blood safety: proportion of blood units screened for blood-borne diseases	100%	100% of blood donations are tested for hepatitis B and C	((59))
Safe injections: proportion of safe healthcare injections	100%	Additional data needed	
Percentage of people living with hepatitis C diagnosed	60%	58.5%	This study
Percentage of people living with hepatitis C diagnosed and cured	50%	Additional data needed	

Abbreviation: PWID, people who inject drugs

^a^ The domestic vaccine coverage goal in Canada is to achieve 90% vaccination coverage by 17 years of age for one or more doses of the hepatitis B vaccine, by 2025 ((60)). As of 2024, no other domestic targets were set

## Discussion

Although the overall viral hepatitis burden is low in the general Canadian population, the 2021 national estimates indicate that certain populations and communities remain disproportionately affected, and that a significant number of people in Canada would benefit from targeted testing and treatment. These estimates mostly use data sources preceding the COVID-19 pandemic. Measuring post-pandemic estimates will be important to account for decreased demand for, and access to prevention, testing, treatment and care services for HBV and HCV. Given changes in methods and available evidence, these estimates replace previously published estimates and should not be compared over time.

It is estimated that 0.68% of people in Canada were living with CHB at the end of 2021. Other estimates for Canada include a 0.4% prevalence (2007–2011) ((61)) and a 0.6% HBsAg prevalence (2022) ((62,63)), which are comparable to the results in this study. Immigrants from countries where HBV is common have the highest burden, by far. Regarding progress towards HBV elimination, we cannot report on the incidence target using available data. Estimates suggest that Canada is close to meeting the 60% diagnosis target for 2025 with 57.5% people living with CHB who were aware of their infection. Awareness data for key populations is scarce, limiting the evidence base for planning targeted interventions. An estimated 0.72 deaths per 100,000 population were identified as HBV-related in 2021, suggesting that the 2025 target of seven per 100,000 population is met, which is supported by a modelling study estimating one per 100,000 population HBV-related deaths in Canada in 2019 ((64)). The HBV vaccine coverage of 89% in adolescents approximates the 2025 target of 90% among children. We estimate that less than 2% of infants born in Canada in 2021 had access to universal birth dose vaccination, far from the 70% global target set for 2025. Of note, health care is a provincial and territorial responsibility, and the *Canadian Immunization Guide* indicates that the HBV vaccine should be provided according to provincial and territorial immunization schedules ((65)). Hepatitis B treatment data were not nationally validated at the time of press.

An estimated 21.47 new HCV infections per 100,000 population occurred in 2021, highlighting that more prevention work, including harm reduction services in all settings ((8,10)), is needed to reduce the incidence of HCV and meet the 2025 target of 13 per 100,000 population. We estimate that 0.99% of the Canadian population was ever infected with HCV. Other estimates include a 0.07% anti-HCV prevalence among Canadian first-time blood donors in nine provinces, representing a low-risk and undiagnosed population ((66)). We estimate the CHC prevalence at 0.56% in the general population; other CHC estimates include a prevalence of viremic infection of 130,000 people (2022) ((67)), and of 0.03% among low-risk undiagnosed blood donors (2021) ((66)). Among people living with CHC, we estimate that 58.5% were diagnosed, suggesting that Canada is close to meeting the 2025 diagnosis target of 60%. However, additional data will be needed to quantify awareness of CHC in all key populations. We identified 2.54 HCV-related deaths per 100,000 population, suggesting that Canada has met the 2025 target of 3 per 100,000. However, this finding contrasts with other evidence suggesting a seven per 100,000 population mortality rate in Canada for 2021 ((64)). Of note, in absolute numbers, more deaths were attributed to HCV than HBV in 2021, despite the availability of curative treatment which is associated with reduced mortality ((68)).Treatment data suggest that more people are being treated for HCV each year than there are new infections. Administrative data for key populations is not available at the national level, although it is documented that self-reported treatment uptake varies by key populations ((32,69)). Since treatment estimates do not consider mortality among treated individuals, we are unable to report on the proportion treated among people living with diagnosed HCV.

### Limitations

The main strengths of this study are the use of updated Canadian Health Measures Survey (CHMS) data, in combination with results from modelling using national surveillance data as model inputs for the first national HBV estimates and updated HCV estimates. We also compiled a thorough list of relevant scientific studies to inform the workbook estimates.

The analyses for this study have several limitations. First, mathematical modelling used for incidence (HCV) and as a lower bound for prevalence was based on reported cases, likely underestimating the true burden. The COVID-19 pandemic had an impact on STBBI testing in Canada ((70–73)), potentially making the modelled estimates for 2020–2021 an underestimation despite mathematical corrections. The workbook method produces large ranges of uncertainty given the heterogeneity of studies (e.g., participants, sampling, methods, geographic location) and data. In some instances, results from local studies were extrapolated nationally. These may impact the precision and accuracy of the estimates. Data is limited for key populations, restricting our capacity to provide estimates for all. Moreover, given the intersection of risks and identities across key populations, estimates cannot be added together or used to create proportions. The general population estimates for CHC rely on data for a reference period up to 2019, therefore this study’s findings may be more representative of the burden for 2019 than for the end of 2021. Measures of HBsAg and HCV RNA, where applicable, were used as proxies for CHB and CHC infections, respectively. Both could potentially result in a slightly overestimated chronicity, given that HBsAg is a marker of active HBV infection that occurs in its acute or chronic phase ((74)), and that HCV RNA is detectable for most people in the acute phase of the infection ((75)). The measure of the HBV vaccine target is likely overestimated since it is measured in adolescents with at least one dose. For mortality, a direct measurement method of documented causes of deaths was used, which excludes all deaths among undiagnosed individuals. As well, potential underreporting of causes on death certification is likely, which could lead to underestimation of the measure towards the target for deaths not identified as viral hepatitis-related. On the other hand, deaths with other direct causes, such as accidents or drug toxicity, may be misclassified as viral hepatitis-related, which would result in overestimation of mortality. Given the nature of this study, we could not control for confounding and missing data.

## Conclusion

Prevention of advanced liver disease caused by viral hepatitis is possible, and epidemiological estimates are essential to identify gaps in the care continuum by key population, contribute to planning evidence-based interventions, and track progress towards elimination as a public health concern. While these estimates suggest that Canada has met or is on track to meet six of the 15 global targets for 2025, more work is needed to address data gaps and to meet the targets that are not on track, such as HCV incidence. Additional national-level data are needed to produce estimates of prevalence and proportion unaware among all key population and to report Canada’s progress towards all elimination targets, including validated treatment data for HBV and cascade of care data.

## References

[1] World Health Organization. Global progress report on HIV, viral hepatitis and sexually transmitted infections, 2021. Accountability for the global health sector strategies 2016–2021: actions for impact. Geneva, CH: WHO; 2021. https://www.who.int/publications/i/item/9789240027077

[2] Paccoud O, Surgers L, Lacombe K. [Hepatitis B virus infection: natural history, clinical manifestations and therapeutic approach]. Rev Med Interne 2019;40(9):590–8. 10.1016/j.revmed.2019.03.333 30982550

[3] World Health Organization. Global hepatitis report 2024: Action for access in low- and middle-income countries. Geneva, CH: WHO; 2024. https://www.who.int/publications/i/item/9789240091672

[4] Yau AH, Marquez-Azalgara V, Yoshida EM. Hepatitis C (chronic). BMJ Clin Evid 2015:0921. https://pmc.ncbi.nlm.nih.gov/articles/PMC4479184/PMC4479184 26107930

[5] Asselah T, Marcellin P, Schinazi RF. Treatment of hepatitis C virus infection with direct-acting antiviral agents: 100% cure? Liver Int 2018;38(Suppl 1 Suppl 1):7–13. 10.1111/liv.13673 29427484 PMC7713514

[6] Shah H, Bilodeau M, Burak KW, Cooper C, Klein M, Ramji A, Smyth D, Feld JJ; Canadian Association for the Study of the Liver. The management of chronic hepatitis C: 2018 guideline update from the Canadian Association for the Study of the Liver. CMAJ 2018;190(22):E677–87. 10.1503/cmaj.170453 29866893 PMC5988519

[7] Canadian Network on Hepatitis C Blueprint Writing Committee and Working Groups. Blueprint to inform hepatitis C elimination efforts in Canada. Toronto, ON: CanHepC; 2019. https://www.canhepc.ca/en/blueprint

[8] Public Health Agency of Canada. Government of Canada’s Sexually transmitted and blood-borne infections (STBBI) Action plan 2024-2030. Ottawa, ON: PHAC; 2024. https://www.canada.ca/en/public-health/services/publications/diseases-conditions/sexually-transmitted-blood-borne-infections-action-plan-2024-2030.html

[9] Public Health Agency of Canada. Reducing the health impact of sexually transmitted and blood-borne infections in Canada by 2030: A pan-Canadian STBBI framework for action. Ottawa, ON: PHAC; 2018. https://www.canada.ca/en/public-health/services/infectious-diseases/sexual-health-sexually-transmitted-infections/reports-publications/sexually-transmitted-blood-borne-infections-action-framework.html

[10] Ontario Hepatitis C Elimination Planning Group, Advisory Committee and Working Groups. The Ontario Hepatitis C Elimination Roadmap. 2023. https://endhepc.ca/wp-content/uploads/2023/03/Ontario-Hepatitis-C-Elimination-Roadmap-Full-Report-in-English.pdf

[11] World Health Organization. Global health sector strategies on, respectively, HIV, viral hepatitis and sexually transmitted infections for the period 2022–2030. Geneva, CH: WHO; 2022. https://www.who.int/publications/i/item/9789240053779

[12] Statistics Canada. Population estimates on July 1, by age and gender, 2001–2023. Ottawa, ON: StatCan; 2024. https://www150.statcan.gc.ca/t1/tbl1/en/tv.action?pid=1710000501&request_locale=en

[13] Popovic N, Williams A, Périnet S, Campeau L, Yang Q, Zhang F, Yan P, Feld J, Janjua N, Klein M, Krajden M, Wong W, Cox J. National Hepatitis C estimates: Incidence, prevalence, undiagnosed proportion and treatment, Canada, 2019. Can Commun Dis Rep 2022;48(11-12):540–9. 10.14745/ccdr.v48i1112a07 38222827 PMC10786238

[14] Smith-Roberge J, Forouzannia F, Hamadeh A, Feng Z, Popovic N, Wong WW. A Mathematical Framework to Estimate Chronic Hepatitis B Prevalence and Undiagnosed Proportion. JMIR Preprints 2024:66309. 10.2196/preprints.66309 PMC12367349 40835390

[15] Newcombe RG. Two-sided confidence intervals for the single proportion: comparison of seven methods. Stat Med 1998;17(8):857–72. 10.1002/(SICI)1097-0258(19980430)17:8<857::AID-SIM777>3.0.CO;2-E 9595616

[16] Statistics Canada. Table 17-10-0016-01: Estimates of births, by gender, annual. Ottawa, ON: StatCan; 2024. https://www150.statcan.gc.ca/t1/tbl1/en/tv.action?pid=1710001601

[17] Public Health Agency of Canada. Highlights from the 2021 childhood National Immunization Coverage Survey (cNICS). Ottawa, ON: PHAC; 2024. https://www.canada.ca/en/public-health/services/immunization-vaccines/vaccination-coverage/2021-highlights-childhood-national-immunization-coverage-survey.html

[18] Statistics Canada. Statistics Canada, Canadian Vital Statistics - Death database (CVSD). Ottawa, ON: StatCan; 2024. https://www23.statcan.gc.ca/imdb/p2SV.pl?Function=getSurvey&SDDS=3233

[19] Trubnikov M, Yan P, Archibald C. Estimated prevalence of Hepatitis C Virus infection in Canada, 2011. Can Commun Dis Rep 2014;40(19):429–36. 10.14745/ccdr.v40i19a02 29769874 PMC5864479

[20] Hansen PC, Pereyra V, Scherer G. Least Squares Data Fitting with Applications: Johns Hopkins University Press; 2013.

[21] Périnet S, Williams A, Yang Q, Campeau L, Day J, Lamboo L, Lee ER, Osiowy C, Popovic N. Prevalence and awareness of hepatitis B and hepatitis C and vaccine-induced immunity to hepatitis B: Findings from the Canadian Health Measure Survey, 2016–2019. Can Commun Dis Rep 2025;51(6/7):238–48. 10.14745/ccdr.v51i67a03 PMC12388309 40880906

[22] Biondi MJ, Marchand-Austin A, Cronin K, Nanwa N, Ravirajan V, Mandel E, Goneau LW, Mazzulli T, Shah H, Capraru C, Janssen HL, Sander B, Feld JJ. Prenatal hepatitis B screening, and hepatitis B burden among children, in Ontario: a descriptive study. CMAJ 2020;192(43):E1299–305. 10.1503/cmaj.200290 33106301 PMC7577574

[23] Adeleye AO, Plitt SS, Douglas L, Charlton CL. Overview of a Provincial Prenatal Communicable Disease Screening Program: 2002-2016. J Obstet Gynaecol Can 2020;42(3):269–76. 10.1016/j.jogc.2019.05.013 31447401

[24] Binka M, Butt ZA, Wong S, Chong M, Buxton JA, Chapinal N, Yu A, Alvarez M, Darvishian M, Wong J, McGowan G, Torban M, Gilbert M, Tyndall M, Krajden M, Janjua NZ. Differing profiles of people diagnosed with acute and chronic hepatitis B virus infection in British Columbia, Canada. World J Gastroenterol 2018;24(11):1216–27. 10.3748/wjg.v24.i11.1216 29568202 PMC5859224

[25] Campeau L, Elliott J, Williams A, Périnet S, Yang Q, Cox J, Feld JJ, Greenaway C, Popovic N. Estimated prevalence of hepatitis B and C among immigrants in Canada. Can Commun Dis Rep 2025;51(6/7):214–22. 10.14745/ccdr.v51i67a01 PMC12372648 40861919

[26] Yasseen AS, Kwong JC, Feld JJ, Kustra R, MacDonald L, Greenaway CC, Janjua NZ, Mazzulli T, Sherman M, Lapointe-Shaw L, Sander B, Crowcroft NS. The viral hepatitis B care cascade: A population-based comparison of immigrant groups. Hepatology 2022;75(3):673–89. 10.1002/hep.32162 34537985

[27] Sorge J, Colyer S, Cox J, Kroch A, Lachowsky N, Popovic N, Yang Q. Estimation of the population size of gay, bisexual and other men who have sex with men in Canada, 2020. Can Commun Dis Rep 2023;49(11-12):465–76. 10.14745/ccdr.v49i1112a02 38504876 PMC10946585

[28] Thompson KA, Blank G, Toy J, Moore DM, Lachowsky N, Bacani N, Zhang W, Sereda P, Lima VD, Barrios R, Montaner JS, Hull MW. Chronic Hepatitis B Infection Among Preexposure Prophylaxis Users Enrolled in a Population-Based Program in British Columbia, Canada. Open Forum Infect Dis 2021;8(11):ofab492. 10.1093/ofid/ofab492 34805433 PMC8598915

[29] Yasseen AS 3rd, Kwong JC, Feld JJ, Janjua NZ, Greenaway C, Lapointe-Shaw L, Sherman M, Mazzulli T, Kustra R, MacDonald L, Sander B, Crowcroft NS. Viral hepatitis C cascade of care: A population-level comparison of immigrant and long-term residents. Liver Int 2021;41(8):1775–88. 10.1111/liv.14840 33655665

[30] Bartlett SR, Yu A, Chapinal N, Rossi C, Butt Z, Wong S, Darvishian M, Gilbert M, Wong J, Binka M, Alvarez M, Tyndall M, Krajden M, Janjua NZ. The population level care cascade for hepatitis C in British Columbia, Canada as of 2018: impact of direct acting antivirals. Liver Int 2019;39(12):2261–72. 10.1111/liv.14227 31444846

[31] Vanderhoff A, Smookler D, Biondi MJ, Enman S, Fuliang T, Mahmood S, Crespi A, Marquez M, Van Uum R, You L, Wolfson-Stofko B, Logan R, LeDrew E, Shah H, Janssen H, Capraru C, Venier E, Feld JJ. Leveraging COVID-19 vaccination to promote hepatitis C screening. Hepatol Commun 2022;7(1):e2101. 10.1002/hep4.2101 36329631 PMC9827963

[32] Tarasuk J, Zhang J, Lemyre A, Cholette F, Bryson M, Paquette D. National findings from the Tracks survey of people who inject drugs in Canada, Phase 4, 2017-2019. Can Commun Dis Rep 2020;46(5):138–48. 10.14745/ccdr.v46i05a07 35283692 PMC8868043

[33] Selfridge M, Barnett T, Lundgren K, Guarasci K, Milne R, Drost A, Fraser C. Treating people where they are: nurse-led micro-elimination of hepatitis C in supported housing sites for networks of people who inject drugs in Victoria, Canada. Public Health Nurs 2022;39(5):1009–16. 10.1111/phn.13092 35537120

[34] Lettner B, Mason K, Greenwald ZR, Broad J, Mandel E, Feld JJ, Powis J. Rapid hepatitis C virus point-of-care RNA testing and treatment at an integrated supervised consumption service in Toronto, Canada: a prospective, observational cohort study. Lancet Reg Health Am 2023;22:100490. 10.1016/j.lana.2023.100490 37388709 PMC10300568

[35] Williams A, Sorge J, Périnet S, Yang Q, Cox J, Bonn M, Smoke A, Popovic N. Estimating the population size of people who inject drugs in Canada. Can Commun Dis Rep 2025; Upcoming. 10.14745/ccdr.v51i09a06 PMC12704403 41403896

[36] Biondi MJ, Hirode G, Capraru C, Vanderhoff A, Karkada J, Wolfson-Stofko B, Smookler D, Friedman SM, Bates K, Mazzulli T, Juan JV, Shah H, Hansen BE, Feld JJ, Janssen H. Birth cohort hepatitis C antibody prevalence in real-world screening settings in Ontario. Can Liver J 2022;5(3):362–71. 10.3138/canlivj-2021-0036 36133900 PMC9473558

[37] Broad J, Mason K, Guyton M, Lettner B, Matelski J, Powis J. Peer outreach point-of-care testing as a bridge to hepatitis C care for people who inject drugs in Toronto, Canada. Int J Drug Policy 2020;80:102755. 10.1016/j.drugpo.2020.102755 32416538

[38] Barnett T, Selfridge M, Mundo L, Johnson S, Drost A, Guarasci K, Lundgren K, Fraser C. Collaborating with prison to test and treat people who use drugs in Victoria, British Columbia. Annual Meeting of the Canadian Association for the Study of the Liver (CASL), the Canadian Network on Hepatitis C (CANHEPC) and the Canadian Association of Hepatology Nurses (CAHN) 2024; February 27–March 3 2024; University of Toronto Press; 2024.

[39] Courtemanche Y, Poulin C, Serhir B, Alary M. HIV and hepatitis C virus infections in Quebec’s provincial detention centres: comparing prevalence and related risky behaviours between 2003 and 2014-2015. Can J Public Health 2018;109(3):353–61. 10.17269/s41997-018-0047-4 29981093 PMC6964431

[40] Kronfli N, Dussault C, Klein MB, Lebouché B, Sebastiani G, Cox J. The hepatitis C virus cascade of care in a Quebec provincial prison: a retrospective cohort study. CMAJ Open 2019;7(4):E674–9. 10.9778/cmajo.20190068 31796509 PMC6890491

[41] Statistics Canada. Table 35-10-0154-01: Average counts of adults in provincial and territorial correctional programs. Ottawa, ON: StatCan; 2024. https://www150.statcan.gc.ca/t1/tbl1/en/tv.action?pid=3510015401

[42] Lydon-Hassen K, Jonah L, Mayotte L, Hrabowy A, Graham B, Missens B, Nelson A, Andkhoie M, Nahachewsky D, Yalamanchili DT, Gupta S, Ndubuka N, Khan I, Yacoub W, Bryson M, Paquette D. Summary findings from Tracks surveys implemented by First Nations in Saskatchewan and Alberta, Canada, 2018-2020. Can Commun Dis Rep 2022;48(4):146–56. 10.14745/ccdr.v48i04a05 35480707 PMC9017804

[43] Mendlowitz AB, Bremner KE, Krahn M, Walker JD, Wong WW, Sander B, Jones L, Isaranuwatchai W, Feld JJ. Characterizing the cascade of care for hepatitis C virus infection among Status First Nations peoples in Ontario: a retrospective cohort study. CMAJ 2023;195(14):E499–512. 10.1503/cmaj.220717 37040993 PMC10089629

[44] Statistics Canada. Table 98-10-0292-01: Indigenous identity population by gender and age: Canada, provinces and territories, census metropolitan areas and census agglomerations. Ottawa, ON: StatCan; 2024. https://www150.statcan.gc.ca/t1/tbl1/en/cv.action?pid=9810029201

[45] Thompson KA, Blank G, Toy J, Moore DM, Lachowsky N, Bacani N, Zhang W, Sereda P, Lima VD, Barrios R, Montaner JS, Hull MW. Prevalence and incidence of hepatitis C infection amongst men who have sex with men in a population-based pre-exposure prophylaxis program in British Columbia, Canada. Liver Int 2022;42(7):1528–35. 10.1111/liv.15237 35274805

[46] Tabatabavakili S, Aleyadeh W, Cerrocchi O, Janssen HL, Hansen BE, Bogoch II, Feld JJ; Viral Hepatitis Care Network Investigators. Incidence of Hepatitis C Virus Infections Among Users of Human Immunodeficiency Virus Pre-exposure Prophylaxis. Clin Gastroenterol Hepatol 2022;20(3):674–81. 10.1016/j.cgh.2021.03.006 33737225

[47] Bolotin S, Feld JJ, Garber G, Wong WW, Guerra FM, Mazzulli T. Population-based estimate of hepatitis C virus prevalence in Ontario, Canada. PLoS One 2018;13(1):e0191184. 10.1371/journal.pone.0191184 29360823 PMC5779675

[48] Forouzannia F, Hamadeh A, Passos-Castilho AM, Erman A, Yu A, Feng Z, Janjua NZ, Sander B, Greenaway C, Wong WW. Impact of new direct-acting antiviral therapy on the prevalence and undiagnosed proportion of chronic hepatitis C infection. Liver Int 2024;44(6):1383–95. 10.1111/liv.15875 38445848

[49] Forouzannia F, Eze N, Clement F, Wong W. The impact of new DAA therapy on the prevalence and undiagnosed proportion of chronic hepatitis C infection in Alberta: a model-based analysis. Annual Meeting of the Canadian Association for the Study of the Liver (CASL), the Canadian Network on Hepatitis C (CANHEPC) and the Canadian Association of Hepatology Nurses (CAHN) 2024; February 27–March 3 2024; University of Toronto Press; 2024.

[50] Pearce ME, Bartlett SR, Yu A, Lamb J, Reitz C, Wong S, Alvarez M, Binka M, Velásquez Garcia H, Jeong D, Clementi E, Adu P, Samji H, Wong J, Buxton J, Yoshida E, Elwood C, Sauve L, Pick N, Krajden M, Janjua NZ. Women in the 2019 hepatitis C cascade of care: findings from the British Columbia Hepatitis Testers cohort study. BMC Womens Health 2021;21(1):330. 10.1186/s12905-021-01470-7 34511082 PMC8436483

[51] Binka M, Janjua NZ, Grebely J, Estes C, Schanzer D, Kwon JA, Shoukry NH, Kwong JC, Razavi H, Feld JJ, Krajden M. Assessment of Treatment Strategies to Achieve Hepatitis C Elimination in Canada Using a Validated Model. JAMA Netw Open 2020;3(5):e204192. 10.1001/jamanetworkopen.2020.4192 32374397 PMC7203608

[52] Hamadeh A, Feng Z, Krahn M, Wong WW. A model-based framework for chronic hepatitis C prevalence estimation. PLoS One 2019;14(11):e0225366. 10.1371/journal.pone.0225366 31751393 PMC6874092

[53] Greenwald ZR, Bouck Z, McLean E, Mason K, Lettner B, Broad J. Integrated supervised consumption services and hepatitis C testing and treatment among people who inject drugs in Toronto, Canada: a cross-sectional analysis. J Viral Hepat 2022. 10.1111/jvh.13780 36461705

[54] Hannigan A. Improving HCV Cascade of Care Among People Who Use Drugs: A Rapid Pilot Program Evaluation and Implication for Future Interventions. British Columbia Centre for Excellence in HIV/AIDS 2023.

[55] Mendlowitz A, Bremner KE, Walker JD, Wong WW, Feld JJ, Sander B, Jones L, Isaranuwatchai W, Krahn M. Hepatitis C virus infection in First Nations populations in Ontario from 2006 to 2014: a population-based retrospective cohort analysis. CMAJ Open 2021;9(3):E886–96. 10.9778/cmajo.20200164 34584007 PMC8486470

[56] Smookler D, Beck A, Head B, Quoquat L, Albany C, Farrell T, Gordon J, Thurston N, You L, Capraru C, McKay M, Kim J, Feld JJ, Shah H. A collaborative approach to hepatitis C testing in two First Nations communities of northwest Ontario. Can Liver J 2022;5(3):329–38. 10.3138/canlivj-2021-0031 36133895 PMC9473560

[57] Rai M, Lowe C, Flemming JA. Screening for hepatitis C in an outpatient endoscopy unit. Can Liver J 2021;4(3):311–6. 10.3138/canlivj-2020-0038 35992259 PMC9202776

[58] Hamadeh A, Haines A, Feng Z, Thein HH, Janjua NZ, Krahn M, Wong WW. Estimating chronic hepatitis C prevalence in British Columbia and Ontario, Canada, using population-based cohort studies. J Viral Hepat 2020;27(12):1419–29. 10.1111/jvh.13373 32810886

[59] O’Brien SF, Drews SJ, Lewin A, Osiowy C, Drebot MA, Renaud C. Canadian blood suppliers: an expanding role in public health surveillance? Can Commun Dis Rep 2022;48(4):124–30. 10.14745/ccdr.v48i04a02 35480705 PMC9017805

[60] Public Health Agency of Canada. Vaccination Coverage Goals and Vaccine Preventable Disease Reduction Targets by 2025. Ottawa, ON: PHAC; 2022. [Accessed 2024 July 1]. https://www.canada.ca/en/public-health/services/immunization-vaccine-priorities/national-immunization-strategy/vaccination-coverage-goals-vaccine-preventable-diseases-reduction-targets-2025.html

[61] Rotermann M, Langlois K, Andonov A, Trubnikov M. Seroprevalence of hepatitis B and C virus infections: Results from the 2007 to 2009 and 2009 to 2011 Canadian Health Measures Survey. Health Rep 2013;24(11):3–13. 24259199

[62] Polaris Observatory Collaborators. Global prevalence, cascade of care, and prophylaxis coverage of hepatitis B in 2022: a modelling study. Lancet Gastroenterol Hepatol 2023;8(10):879–907. 10.1016/S2468-1253(23)00197-8 37517414

[63] Biondi M, Marchand-Austin A, Mandel E, Goneau L, Mazzulli T, Janssen H. Prenatal HBsAg screening rates and follow-up DNA testing low in Ontario. Can Liver J 2019;2(2):90–1. 10.1503/cmaj.200290 33106301 PMC7577574

[64] Institute for Health Metrics and Evaluation. GBD Results. Seattle, WA: IHME; 2021. [Accessed 2024 April 1]. https://vizhub.healthdata.org/gbd-results/

[65] Public Health Agency of Canada. Hepatitis B vaccines: Canadian Immunization Guide. Ottawa, ON: PHAC; 2022. https://www.canada.ca/en/public-health/services/publications/healthy-living/canadian-immunization-guide-part-4-active-vaccines/page-7-hepatitis-b-vaccine.html

[66] O’Brien SF, Ehsani-Moghaddam B, Osmond L, Fan W, Goldman M, Drews SJ. Epidemiology of Hepatitis C over 28 years of monitoring Canadian blood donors: insight into a low-risk undiagnosed population. BMC Public Health 2024;24(1):2319. 10.1186/s12889-024-19790-2 39192303 PMC11348590

[67] The Polaris Observatory. Countries/Territories — Dashboard for Canada. 2024. https://cdafound.org/polaris-countries-dashboard/

[68] Carrat F, Fontaine H, Dorival C, Simony M, Diallo A, Hezode C, De Ledinghen V, Larrey D, Haour G, Bronowicki JP, Zoulim F, Asselah T, Marcellin P, Thabut D, Leroy V, Tran A, Habersetzer F, Samuel D, Guyader D, Chazouilleres O, Mathurin P, Metivier S, Alric L, Riachi G, Gournay J, Abergel A, Cales P, Ganne N, Loustaud-Ratti V, D’Alteroche L, Causse X, Geist C, Minello A, Rosa I, Gelu-Simeon M, Portal I, Raffi F, Bourliere M, Pol S; French ANRS CO22 Hepather cohort. Clinical outcomes in patients with chronic hepatitis C after direct-acting antiviral treatment: a prospective cohort study. Lancet 2019;393(10179):1453–64. 10.1016/S0140-6736(18)32111-1 30765123

[69] Lambert G, Apelian H, Tremblay C, Mbaye R, Pardoe W, Dvorakova M. Engage Montréal, Portrait de la santé sexuelle des hommes de la région métropolitaine de Montréal ayant des relations sexuelles avec des hommes, Cyle 2017-2018 Recueil de tableaux et outils. Direction générale de santé publique du CIUSSS du Centre-Sud-de-l’Île-de-Montréal. 2022. https://www.inspq.qc.ca/sites/default/files/documents/itss/engage_faitssaillants_mars-2019-b.pdf

[70] Mandel E, Peci A, Cronin K, Capraru CI, Shah H, Janssen HL, Tran V, Biondi MJ, Feld JJ. The impact of the first, second and third waves of covid-19 on hepatitis B and C testing in Ontario, Canada. J Viral Hepat 2022;29(3):205–8. 10.1111/jvh.13637 34820967

[71] Binka M, Bartlett S, Velásquez García HA, Darvishian M, Jeong D, Adu P, Alvarez M, Wong S, Yu A, Samji H, Krajden M, Wong J, Janjua NZ. Impact of COVID-19-related public health measures on HCV testing in British Columbia, Canada: an interrupted time series analysis. Liver Int 2021;41(12):2849–56. 10.1111/liv.15074 34592046 PMC8662267

[72] Public Health Agency of Canada. Survey on the impact of COVID-19 on the delivery of STBBI prevention, testing and treatment, including harm reduction services, in Canada. Ottawa, ON: PHAC; 2021. https://www.canada.ca/en/public-health/services/publications/diseases-conditions/survey-impact-covid-19-delivery-stbbi-prevention-testing-treatment.html

[73] Kronfli N, Leone F, Dussault C, Miliani G, Gallant E, Potter M, Cox J. Impact of the COVID-19 pandemic on hepatitis C virus screening in provincial prisons in Montreal, Quebec, Canada. Front Public Health 2024;12:1380126. 10.3389/fpubh.2024.1380126 39109158 PMC11302355

[74] Coffin CS, Fung SK, Alvarez F, Cooper CL, Doucette KE, Fournier C, Kelly E, Ko HH, Ma MM, Martin SR, Osiowy C, Ramji A, Tam E, Villeneuve JP. Management of Hepatitis B Virus Infection: 2018 Guidelines from the Canadian Association for the Study of Liver Disease and Association of Medical Microbiology and Infectious Disease Canada. Can Liver J 2018;1(4):156–217. 10.3138/canlivj.2018-0008 35992619 PMC9202759

[75] Glynn SA, Wright DJ, Kleinman SH, Hirschkorn D, Tu Y, Heldebrant C, Smith R, Giachetti C, Gallarda J, Busch MP. Dynamics of viremia in early hepatitis C virus infection. Transfusion 2005;45(6):994–1002. 10.1111/j.1537-2995.2005.04390.x 15934999

